# Bi-logistic model for disease dynamics caused by *Mycobacterium tuberculosis* in Russia

**DOI:** 10.1098/rsos.171033

**Published:** 2017-09-13

**Authors:** Anastasia I. Lavrova, Eugene B. Postnikov, Olga A. Manicheva, Boris I. Vishnevsky

**Affiliations:** 1 Saint-Petersburg State University, Medical Faculty, Universitetskaya emb., 7/9, Saint-Petersburg, Russia; 2 Saint-Petersburg State Research Institute of Phthisiopulmonology, Lygovsky avenue 2-4, Saint-Petersburg, Russia; 3 Department of Theoretical Physics, Kursk State University, Radishcheva street 33, Kursk, Russia

**Keywords:** *Mycobacterium tuberculosis*, virulence, mixed subpopulations, compartmental model

## Abstract

In this work, we explore epidemiological dynamics by the example of tuberculosis in Russian Federation. It has been shown that the epidemiological dynamics correlates linearly with the virulence of *Mycobacterium tuberculosis* during the period 1987–2012. To construct an appropriate model, we have analysed (using LogLet decomposition method) epidemiological World Health Organization (WHO) data (period 1980–2014) and obtained, as result of their integration, a curve approximated by a bi-logistic function. This fact allows a subdivision of the whole population into parts, each of them satisfies the Verhulst-like models with different constant virulences introduced into each subsystem separately. Such a subdivision could be interconnected with the heterogeneous structure of mycobacterial population that has a high ability of adaptation to the host and strong mutability.

## 1. Introduction

Tuberculosis remains a main problem for public health resulting in new TB cases and deaths [1] due to high development of multi- and extensively drug resistant (MDR and XDR) *Mycobacterium tuberculosis* (*MbT*). The scale of the tuberculosis epidemic with multi-drug resistance (MDR-TB) is huge; according to the World Health Organization (WHO) report, 450 000 patients with MDR-TB were registered in 2012 year. Also, according to the WHO, in 92 countries were registered the cases with extensively drug-resistant TB (XDR-TB) that is the most hard form of disease leading to the lethal outcome. As was reported by the Department of Public Health and Communications of Ministry of Healthcare of the Russian Federation, the incidences with sensitive form of tuberculosis decreased in 2015 comparing with 2014, but MDR-TB cases were observed to rise among patients for those whose tuberculosis was detected for the first time. Thus, MDR-TB incidence increased from 4.6 to 5.2 per 100 thousand of population, and the proportion of patients with MDR-TB among persons with bacterial excretion at pulmonary tuberculosis was 47.3%. At the same time, the bacterial load of respiratory material of patients with MDR/XDR-TB does not differ from that in the drug sensitivity tuberculosis, indicating the viability of the pathogen, even with a wide range of its resistance to anti-TB drugs and high epidemiological risks of such strains [2].

Mathematical modelling of tuberculosis development and expansion could be helpful at the diagnosis and prognoses at the TB treatment on the population-level (for review, see [3]). The basis for most models recently developed is the SIR model [4] which includes different types of subpopulations (susceptible, infected and recovered) and describes transmission dynamics between them. There are relatively simple models taking into account uninfected, latent TB and active TB persons and more detailed models that include subdivisions to TB strains: drug sensitive and drug resistant allowing to describe different ways for the infection and the recovery in population in different countries [3,5–8].

To describe a trend in tuberculosis mortality using data for a long period of time, Holloway *et al.* [9] have proposed an approach based on the data fitting by logistic curves without introduction of any detailed mathematical model. Such technique allowed to characterize the dynamics of tuberculosis decline and to show that social aspects in TB prevention, namely, nutrition, hygiene, education about TB, etc., have an impact on reducing the incidence of tuberculosis in a population in most countries and could be a serious strategy in the fight against the disease.

At the same time, a not so long-term dynamics, which has non-monotone character, is still a very challenging problem. One of the key questions here is not only social but microbiological factors affecting such dynamics as well as their combination (for review, see [10,11]). In fact, the key parameter, a knowledge of which allows the conclusion, whether or not an epidemic outbreak occurs, is the basic reproduction number $R_{0}$ [12]. It should be noted that its variation can be closely connected with the actual instant virulence of a pathogen [13,14] which defines its ability of adaptation to a host organism and higher probability to cause a disease. It evolves in dependence on the sensibility or resistance of macroorganism to infection. Therefore, in the scale of epidemic outbreak, this microorganism’s property could be an important factor, which can influence the disease rate, its maximal strength and decrease [15]. In particular, an extended SIR model [14] of malaria developed recently considers the virulence as a phenomenological parameter included in the contact rate and the death rate induced by this disease. Moreover, this problem can be stated as a problem of pathogens evolution with respect to their virulence [16].

Thus, the primary goal of our work is to reveal a possible time-dependent functional composition which adequately approximates actual time dynamics of tuberculosis outbreak in Russia during last three decades reflected in WHO data; to discuss a model, which provides such dynamics, as well as possible control factors, which govern the multimodal TB epidemic activity in Russia detected over several last decades; as well as to discuss an origin of the same, qualitatively noted for *M. tuberculosis* in laboratory studies of the same period.

## 2. Model and method

First of all, we need to determine a class of functional dependencies, which are suitable for the data fitting. As a most natural candidate we consider logistic curves, which can be obtained as certain approximate solutions of the the three-compartmental Kermack–McKendrick SIR model [4]

2.1 $$\begin{array}{rl} \frac{dS}{dt} & =-kSI, \end{array}$$

2.2 $$\begin{array}{rl} \frac{dI}{dt} & =kSI-\tau^{-1}I \end{array}$$

2.3 $$\begin{array}{rl} \;\mathrm{and}\;\frac{dR}{dt} & =\tau^{-1}I, \end{array}$$

where *S* and *I* mean the number of susceptible and infected persons, respectively. The constant *k* is the parameter determined by the infection properties, e.g. by virulence of *MbT* strains, and social (a contact rate) reasons.

The variable *R*, which can be defined as

2.4 $$R(t)=\tau^{-1}\int_{0}^{t}I(t^{\prime })\;dt^{\prime }$$

can be interpreted as proportional to the cumulative number of persons suffering from the infection during the time interval [0,*t*]. Note that we do not connect this variable with a number of recovered/removed persons, as has been done in the original model interpretation, but rather as a generalized number of persons who cannot be further considered as susceptible to *primary* TBC infection.

In fact, we will use below the data (WHO and laboratory data) obtained for patients with tuberculosis detected for the first time, which usually is accompanied by active bacterial excretion to environment. Therefore, the main goal of the hospital’s treatment is to stop an excretion and then the patient is transferred to out-patient treatment. After cessation of bacterial excretion, the patient is either completely cured—in this case, complete decomposition of the inflammation foci or fibrosis of the destruction areas occur in the lungs—or passes into the state of the chronic process, which is characterized by the unclosed inflammation foci that can result in the tuberculosis relapse. The relapse is not considered as first time detected. Whence, the parameter *τ* is attributed to the average duration of an active phase of tuberculosis, as it is conventionally interpreted while using SIR model and its generalizations for TB modelling [7,8,12]. Its combination with the parameter *k*, described above, gives the basic reproduction number $R_{0}=k\tau$, the value of which $R_{0}>1$ corresponds to an emergence of the illness outbreak.

The system (2.1)–(2.3) is supplied with the conservation law

2.5 S+I+R=const.

that corresponds to the assumed constant population size during the time range considered in this study. Another invariant directly follows from the mathematical structure of (2.1)–(2.3) as [17] dividing equation (2.1) by equation (2.3) and then integrating the resulting equation.

2.6 $$\log\;\left(\frac{S}{S_{0}}\right)+k\tau R=0,$$

where *R*_0_=*R*(0)=0 at the very start of an epidemic.

As a result, the system (2.1)–(2.3) with (2.5)–(2.6) substituted, reduces to one nonlinear ordinary differential equation

2.7 $$\frac{dR}{dt}=\tau^{-1}[1-R-S_{0}\;e^{-k\tau R}].$$

Following Kermack & McKendrick [4], it is possible to introduce the expansion

$$e^{-k\tau R}\approx 1-k\tau R+\frac{{\left(k\tau R\right)}^{2}}{2},$$

which is sufficiently accurate for realistic medically observed values of the product *kτ* (see, for example, the discussion of applicability of such simplification to modelling plague and influenza outbreaks in [17]).

However, in contrast to the original [4], where an arbitrary *S*_0_=*S*(0) is considered that is used now as a conventional approach [18], we use the normalization *const*.=1 in equation (2.5) and take into account that *I*_0_≪*S*_0_, i.e. *S*_0_≅1 for this norm. As a result, equation (2.7) reduces to Verhulst’s logistic equation

2.8 $$\frac{dR}{dt}=\tau^{-1}(k\tau -1)R\;\left[1-\frac{{\left(k\tau \right)}^{2}R}{2(k\tau -1)}\right].$$

Such approach has several advantages useful for the analysis of realistic medical data. It operates with lesser number of adjustment constants that results in better stability of numerical algorithms; the solution of equation (2.8) is a relatively simple logistic function, in contrast to the conventional case of an arbitrary *S*_0_ [4,17], which requires the hyperbolic tangent function.

At the same time, this reduced representation keeps the basic features, which are required to reproduce an epidemic outbreak: equation (2.8) has two stationary points: the unstable *R*_*s*1_=0 and the stable *R*_*s*2_=2(*kτ*−1)/(*kτ*)^2^. As a result, *R* takes its values within the interval *R*∈[*R*(0)>0,*R*_*s*2_] and monotonously grows since d*R*/d*t*≥0 within this interval.

In particular, the second derivative

$$\frac{d^{2}R}{dt^{2}}=\tau^{-1}(k\tau -1)\frac{dR}{dt}\;\left[1-\frac{{\left(k\tau \right)}^{2}R}{\left(k\tau -1\right)}\right]$$

changes its sign when *R*=*R*_*s*2_/2, which implies that *R* curve has an inflection point and the number of actively infected persons *I* has a unique maximum there due to its definition as a derivative of *R*, see (2.3). Thus, a shape of outbreak will be described.

We need to note that one needs to take a non-zero (although it can be arbitrary small) initial condition *R*_0_=*R*(0) to solve (2.8). It is a consequence of the simplification applied to the exact equation (2.7). However, this fact does not prevent usage of the logistic curve

2.9 $$R=\frac{\kappa_{i}}{1+\exp(-\log(81)(t-b)/a)},$$

which is the solution of equation (2.8) for the fitting epidemic curves, since the exact starting time for a widespread illness may be indefinite from medical statistics. Here the parameters are

$$a=\frac{\log(81)}{\tau^{-1}(k\tau -1)},\;\kappa^{-1}=\frac{{\left(k\tau \right)}^{2}R_{0}}{2(k\tau -1)}\;\text{and}\;b=\frac{a\;\log((\kappa /R_{0})-1),}{\log(81)}$$

and the factor log⁡(81) is used to keep correspondence to the work [19], which is supplied by the data processing software, which we will use for the primary fitting our data.

**Figure 1.**
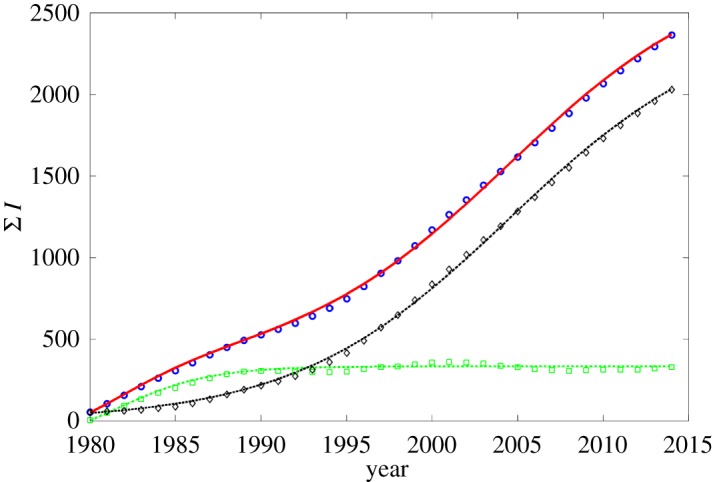
The dynamics of the cumulative incidence of tuberculosis per 100 000 for Russia accordingly to WHO/Europe (circles) and its bi-logistic approximation (red curve). The black curve/diamond markers and green curve/square markers show the decomposition into partial components of the approximation and the data. The parameters fitted: *d*_1_=−190, *a*_1_=12, *b*_1_=1981.5, *κ*_1_=525; *d*_2_=0, *a*_2_=27.5, *κ*_2_=2480, *b*_2_=2004.5.

Thus, we will use further the logistic function as main building blocks for the analysis of recorded medical statistics. But these data (see blue circles in figure 1; their origin will be described in detail below) differ from a single pure logistic growth. For this reason, we will apply the so-called LogLet transform proposed in the work [19] for the decomposition of complex non-stationary growth processes into a series of saturated partial components. Such an approach was proposed in the work cited for a decomposition of the data representing some economic process, such as a sequential replacement of product models on a market. This situation has a certain similarity to the case of change of prevailing microbial strains presented among a population within the concept of so-called ‘Evolution of microbial markets’ [20].

If the full cumulative number of people suffering from the infection will be considered as composed by several subgroups subjected to different *k*_*i*_, *τ*_*i*_, then the total evolution of *R*(*t*) can be represented as a sum of solutions (2.9) supplied with indices *i* denoting individual logistic components,

2.10 $$R=\sum_{i}R_{i}=\sum_{i}\;\left[\frac{\kappa_{i}}{1+\exp(-\log(81)(t-b_{i})/a_{i})}+d_{i}\right],$$

where *d*_*i*_ is determined by the choice of initial background epidemic level mentioned above.

Note also that the standard basic solutions (2.9) allow the coordinate alteration, which gives a straight line in semi-logarithmic coordinates

$$\log(FR_{i}(t))=\frac{\log(81)}{a_{i}}(t-b_{i}),$$

which can be obtained denoting *F*_*i*_(*t*)=*R*_*i*_(*t*)/*κ*_*i*_ in the fraction above and expressing the logarithmic term in the denominator there as

$$FR_{i}=\frac{F_{i}(t)}{1-F_{i}(t)}.$$

Being applied to the full processed data $R=\sum_{i}R_{i}$, this representation as a fraction is called the Fisher–Pry transform. If inflection points of all individual components are sufficiently separated in time, the multimodal growth curve will be transformed into a set of linear sub-intervals, each of which correspond to the individual logistic component of equation (2.10) [19].

## 3. Data and results of their processing

All data explored were taken from the European health for all database (HFA-DB) presented on WHO/Europe’s portal [21]: incidence of tuberculosis per 100 000 for Russia within the time range 1980–2014. They are shown in figure 1 as blue circles. To evaluate their LogLet decomposition, the two-stage numerical procedure was used. At the first step, we applied the specialized online software Loglet Lab 3.0 [22] to obtain first estimations for all coefficients in the series (2.10). The input data were represented as the cumulative sum

$$R_{j}=\sum_{n}^{j}I_{n},$$

replacing the continual integral representation (2.4), where the index *j* denotes a time point of the current time *t*_*j*_.

At the second step, the obtained coefficients *d*_*i*_, *a*_*i*_, *b*_*i*_ *k*_*i*_ were additionally adjusted pointwise to the processed data. The final values obtained at this stage are given in the caption to figure 1, which shows the function (2.10) with these parameters as the red solid curve. One can note quite accurate visual reproduction of the data dynamics that is confirmed by the statistical analysis: the correlation coefficient between actual and fitted data is equal to 98.8%.

Figure 1 demonstrates also two individual components of (2.10): green and black dashed lines correspond to indices *i*=1 and *i*=2, respectively, as well as the decomposition of the processed data into the partial processes obtained as difference of the total *R*_*j*_ and the complementary partial fitted components (they are shown as markers).

**Figure 2.**
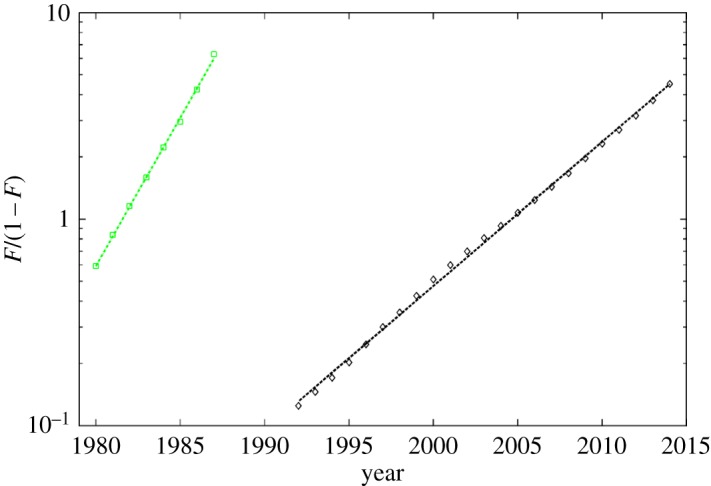
The Fisher–Pry LogLet transform of the data presenting cumulative incidence of tuberculosis per 100 000 for Russia in semi-logarithmic scale. The notation of partial components is the same as in figure 1.

One can see that these partial processes actually resemble logistic growth with saturation (the correlations between the component’s data and their fits are 98.47% and 99.97% from these components.) This conclusion is supported by the Fisher–Pry transform too (figure 2): both components are fitted by straight lines. Moreover, this representation allows clear distinguishing between the time ranges for the prevalence of each partial logistic process.

This time-separated linear components show the coexistence time period between components is quite small. This behaviour may originate from the fact that tuberculosis is a persistent infection widely represented in population but subjected to mutations of *MbT* that correspond to the larger values of the parameters *a*_2_ and *κ*_2_ in comparison with *a*_1_ and *κ*_1_ characterizing ‘old traditional’ tuberculosis. The former is introduced into the population relatively recently and is more aggressive, while the dynamics of previously prevailing strain is practically saturated, see the green curve in figure 1. Some medical evidence of these conclusions will be given in the next section.

Thus, the results of the direct bi-logistic fitting medically recorded data argue that we deal with a replacement of the main infection agent rather than with the competitive process.

**Figure 3.**
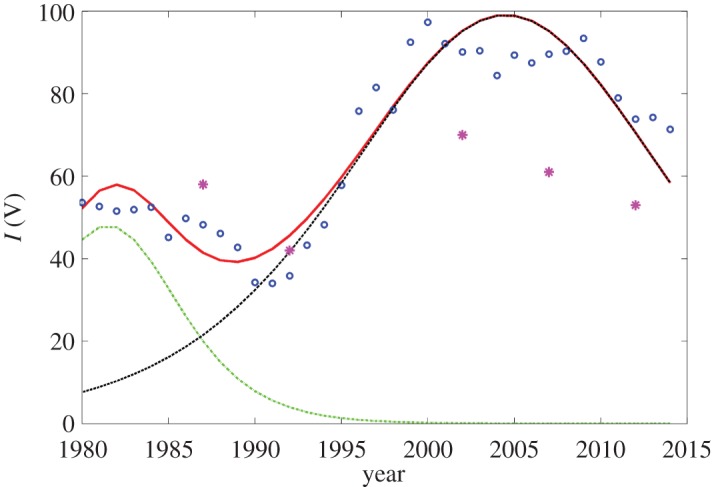
The dynamics of the incidence of tuberculosis per 100 000 for Russia: the actual data accordingly to WHO/Europe (circles) and their bi-logistic model (solid red curve) as well as its two partial components (dashed green and black curves). The asterisks show the known experimental virulence data.

Finally, figure 3 presents the dynamics model incidences obtained via the definition (2.3) with the substituted fitting function (2.10). One can see that it catches the principal bimodal character of the actual data. Owing to the construction of the model, these feature can be explained as a presence of two sequential outbreaks. The first one practically decays within the considered time interval, while the second one only started before 1980 and reached its maximum around 2005.

## 4. Discussion and conclusion

The data processing of TB incidences evaluated based on the bi-logistic combination of the simplified SIR models shows that this models reproduces the observed time dynamics with sufficiently high accuracy. It reveals the detailed background of the non-monotonic dynamics as a coexistence of two SIR processes with a time lag and different basic reproduction numbers $R_{0}^{\left(1\right)}=k_{1}\tau_{1}=1.0019$ and $R_{0}^{\left(2\right)}=k_{2}\tau_{2}=1.0099$. That both values are close to unity corresponds to a persistent character of TBC among the population, but their slight overcoming of unit value leads to long-time acting outbreaks. $R_{0}^{\left(2\right)}>R_{0}^{\left(1\right)}$ and figure 3 shows that the second outbreak, which started in the late 1970s (see black dashed line in figure 3) and presents up to date is more strong (compare green and black dashed curves) in comparison with that one, which reached its maximum in early 1980s (see green line).

It should be pointed out that such transient behaviour is not unique among the time courses of infectious diseases revealed by mathematical modelling. For example, a study [23] of the classic example of localized epidemic outbreak, the plague in Eyam village in 1665–1666, shows the picture quite similar to our figure 1, which has been interpreted as a two-phase process, where the second phase corresponded to the more deadlier (i.e. more virulent) form of the disease. This also allows the discussion of the change in virulence data of TB obtained in laboratory investigations (they are shown in figure 3 as asterisks).

It should be noted that experimental data on the correlation between strains with high and low virulence were obtained using various models, both in animals (subcutaneous, intracranial infection of guinea pigs and also intravenous infection of mice) and on the THP-1 cell line. Each value (asterisks in figure 3) is the percentage of strains with high virulence with respect to the total number of strains of the pathogen investigated during this period; these data have been obtained from patients of the clinics of the St Petersburg Research Institute of Phthisiopulmonology in 1987–2012.

Recently, it is argued [13] that the pathogen virulence has a multifaceted interconnection with the values of the basic reproduction number $R_{0}$ since, in particular, the growing virulence can result in slower recovery from infection and increased probability of a contact transmission. In the case of tuberculosis, the low effectiveness of therapy (39.1%) in patients with multiple and broad drug resistance (MDR and XDR) of the pathogen leads to the same consequences (slower recovery from infection and increased probability of contact transmission) [24].

Moreover, in addition to the classic coevolution model of host–virus interaction [25] (see also [10]), which connects virulence and $R_{0}$ by a nonlinear functional dependence and assumes maximizing virulence as maximizing the basic reproduction number, modern studies consider competitive population dynamics and coevolution of virulent agents in host populations composed by several groups [26].

It should be noted that tuberculosis is the result of the dynamic and multiple-factor interaction of the host and the pathogen with specific genetic and phenotype characteristics for each of them. At the same time, the virulence of a particular strain population isolated from a specific host (macroorganism) during disease is determined by the realization of the pathogenic potential of the cells of this population, their adaptation to the internal environment of the given host. The host–pathogen relationships are realized at different stages: from molecular (genes, proteins, receptors, etc.) to population level. Each level has its own spatial volume and duration of interaction of macro- and microorganisms, while each subsequent level is characterized by the extension of time and space. For example, adhesion of *M. tuberculosis* on the surface of a human cell caused by the interaction of surface molecules of the cell membrane of the pathogen and receptors of the epithelium of the respiratory tract or macrophages is carried out simultaneously and in a volume determined by the molecular level. Bacteria phagocytosis, inflammation and then destruction in the lung or other tissue and finally systemic changes in macro-organism can last over a long period of time (days–months). Coevolution, or interaction between the population of strains of the pathogen circulating in a certain region and the host population, characterized by certain socio-, geo- and ethnic characteristics, continues for decades; *Homo sapiens–Mycobacterium tuberculosis* relationship lasts a thousand years. Of course, the host–pathogen relationships are realized at all levels simultaneously, where all levels interact in a mutual manner. Hence, we would like to clarify that the definition of ‘virulence’ is more correct to apply for the host–pathogen relationship only at the level of the macroorganism/pathogen, but for the population level, which is studied by the methods of molecular epidemiology, it would be better to suggest the concept of pathogen virulent potential of phylogenetic line/subpopulation/cluster. It should be emphasized that the host–pathogen relationship in tuberculosis is affected by the geno- and phenotypic heterogeneity of the populations of both players of this process [27,28].

This is our case, when two subpopulations are considered susceptible to two possible groups of mycobacteria characterized by different virulence reflected in $R_{0}^{\left(1\right)}$ and $R_{0}^{\left(2\right)}$ and extinction of the first group influence in the mid-1990s. The second of these groups may be a cluster of strains of *M. tuberculosis* belonging to the Beijing genetic line, one of the most representative lines on the territory of the Russian Federation. In the years 2004–2006, their population reached 44.4% in the Pskov region [29], 34.0% in central Russia [30], 50.0% in Tuva republic [31], 71.0% in Eastern Siberia [32]. In general, members of this family are distinguished by a high transmission [33], association with multi-drug resistance [34,35] and the ability to cause disease, where it should be noted that the variability of pathogenic characteristics are associated with genetic heterogeneity of strains within bacterial subpopulations [36]. The most successful clone (subline) of the Beijing family on the territory of Russia is B0/W148 [37,38]. For example, in the territory of the Republic of Karelia has been observed an insignificant decrease of the strains fraction of the Beijing family from 67.0% in 2006 to 55.1% in 2012, while the fraction of clonal cluster B0/W148 has increased from 22.9% to 34.9%, respectively. In St Petersburg (the neighbouring region), these ratios remained unchanged. At the same time, the strains of the B0/W148 cluster were 18.0% (2006) and 37.6% (2012) of the number of drug-resistant strains isolated in the territory Karelia. A high degree of association of this subline with drug resistance has been obtained also for strains in St Petersburg [37,39].

Note, respectively, that the ratio of these subgroups (black and green lines in figures 1–3) presented in the whole population changes with time that gives time-varying data for the laboratory determined virulence [40]. Thus, such subdivision opens the question for more detailed characterization and classification of pathogens and subjects of their action in microbiological studies that can be considered as a future perspective challenge.

## Data accessibility

There is no supporting material or special data since all equations, parameters used and algorithm are presented in the main text of the work and could be reproduced by anyone. Thus, the paper contains complete self-sufficient information and does not need any additional data files.
